# Effects of macrophage polarization on gold nanoparticle-assisted plasmonic photothermal therapy†

**DOI:** 10.1039/d1ra03671h

**Published:** 2021-07-19

**Authors:** Hala R. Ali, Salah A. Selim, Daniel Aili

**Affiliations:** Department of Bacteriology and Immunology, Animal Health Research Institute (AHRI), Agriculture Research Center (ARC) Dokii-Giza, P.O. Box 12618 Egypt alihala312@gmail.com; Department of Microbiology, Faculty of Veterinary Medicine, Cairo University Giza Cairo Egypt; Laboratory of Molecular Materials, Division of Biophysics and Bioengineering, Department of Physics, Chemistry and Biology, Linköping University SE-581 83 Linköping Sweden daniel.aili@liu.se

## Abstract

Tumor associated macrophages (TAM) are key pathogenic factors in neoplastic diseases. They are known to have plasticity and can polarize into two opposing phenotypes, including the tumoricidal M1 and the protumoral M2 phenotypes with high prevalence of M2-phentoypes in patients with poor prognosis. Strategies for targeting M2-TAM may consequently increase the efficacy of therapeutic strategies for cancer treatment. Gold nanorod-assisted plasmonic photothermal therapy (PPTT) has emerged as a promising treatment for cancer but the effects of macrophage polarization parameters in the performance of this new treatment modality is still unknown. Herein, human monocytic THP-1 cells were polarized into two opposite phenotypic macrophages (M1-TAM and M2-TAM) and their response to PPTT was examined. M2-TAM exhibits a three-fold increase in AuNP uptake compared to M1-TAM. Laser irradiation results in selective killing of pro-tumoral M2-TAM after treatment with AuNPs with limited effects on anti-tumoral M1-TAM. A positive correlation between the expression of CD206 marker and the AuNP uptake may indicate the role of CD206 in facilitating AuNP uptake. Our findings also suggest that the differences in AuNP avidity and uptake between the M1-TAM and M2-TAM phenotypes may be the rationale behind the effectiveness of PPTT in the treatment of solid tumors.

## Abstract 2

A preferential uptake of gold nanoparticles by macrophages with a protumoral M2 phenotype result in efficient killing upon laser irradiation while keeping M1 phenotypes relatively undamaged.

## Graphical abstract

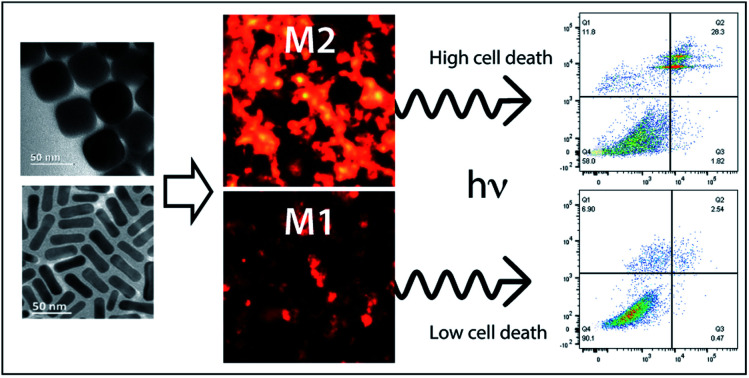

## Introduction

Plasmonic photothermal therapy (PPTT) is a promising technique for combating solid tumors. PPTT exploits plasmonic nanoparticles (NPs) with high extinction cross-section that absorb light, typically in the near infrared range, and convert it into sufficient heat to kill cancer cells.^1,2^ PPTT is dependent on efficient accumulation of the NPs in the tumor microenvironment (TM). The main driving force for accumulation of NPs in solid tumors has been ascribed to passive diffusion through fenestrations between endothelial cells in the tumor vasculature, the so-called enhanced permeation and retention (EPR) effect.^3,4^ Recent findings, however, indicate that active trans-endothelial mechanisms contribute substantially to NP accumulation in tumors.^5^ In addition, the TM constitutes a wide variety of nonmalignant stromal cells, such as macrophages (Mfs),^6^ that also may be involved in NP tumor accumulation. It is however not known to what extent Mfs contribute to and influence the efficacy of PPTT.

**Fig. 1:**
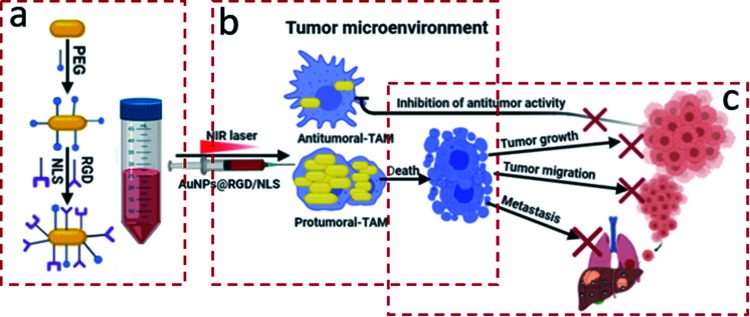
(a) AuNPs were PEGylated and functionalized with RGD and NLS peptides to generate AuNPs@PEG/RGD/NLS. (b) Tumor associated macrophages (TAM) with a protumoral M2 phenotype show substantially larger nanoparticle uptake than antitumoral M1 macrophages. (c) Plasmonic photothermal therapy (PPTT) using AuNPs@PEG/RGD/NLS selectively targets and kill protumoral TAM, which can contribute to blocking of tumor progression.

Tumor associated Mfs (TAM) are the major non-malignant cell population associated with solid tumors.^7^ Mfs are also the primary host recognition system that is responsible for nanoparticle clearance. Mfs are primarily derived from monocyte precursors and can further differentiate into two opposite functional phenotypes in response to microenvironmental cues. The polarization of Mfs is a key pathogenic feature in inflammatory and neoplastic diseases.^8–10^ The classically activated M1-Mfs exhibit anti-tumorigenic activity, and in contrasts the alternative activated M2-Mfs exhibit pro-tumorigenic activity. M2-Mfs are very similar to M2-TAM, and their role in promoting tumor progression to malignancy has been previously documented in multiple studies.^11,12^ M2-TAM is the major type of immune cells of the TM in several types of cancers. The close communication between M2-TAM and cancer cells support intravasation of cancer in blood vessels and consequently in metastasis. Accumulating evidence from previous studies indicate a strong association between a high prevalence of M2-TAM in the tumor microenvironment and poor prognosis outcomes.^13^ For example, a recent study demonstrated that M2-TAM polarized Mfs are highly enriched in gastric cancer and promotes migration of gastric cancer cells either *in vitro* or *in vivo*.^14^ Because of the involvement of the different macrophage phenotypes in the TM, possibilities of detecting or modulating macrophage response could offer a therapeutic or diagnostic advantage. A number of experimental studies investigated the impact of phenotypic difference of Mfs in nanoparticles clearance. Hoppstädter *et al.* indicated that the M2 polarized Mfs promote silica nanoparticle internalization.^15^ Jones *et al.* demonstrated that mouse strains that are prone to Th2 immune responses, which are characterized by prevalence of M2-Mfs, clear nanoparticles at a higher rate than Th1-prone mice with predominantly M1-Mfs.^16^ The effect of macrophage polarization on NP uptake, and consequently their potential role in influencing the efficacy of PPTT in solid tumors, is not known. In our prior research, PPTT has shown great efficacy in curing spontaneous mammary gland tumors in large animal models without any evidence of recurrence or metastasis.^17^ We hypothesize that the different capacity of the two Mfs subtypes that infiltrate the TM to internalize NPs might be the rationale behind the successful treatment. In the present work, we have studied the influence of Mfs polarization on the uptake and efficacy of PPTT using gold nanoparticles (AuNPs) functionalized with polyethylene glycol (PEG) and the integrin targeting peptide RGDRGDRGDRGDPGC (RGD) and nuclear localization signal peptide CGGGPKKKRKVGG (NLS) as illustrated in Fig. 1. Two different shapes of AuNPs were used, gold nanocubes (AuNCs) and gold nanorods (AuNRs). We used a combination of cytokines to generate three different subsets of macrophages from the human monocytic THP-1 cells. Resting cells (M0-Mfs), which were not stimulated, were treated with LPS/IFNγ and IL-4/IL-13 to generate M1-Mfs and M2-Mfs phenotypes, respectively. The AuNP uptake by the two differently polarized Mfs representing the M1-TAM and M2-TAM was examined and the results show significantly higher uptake capacity by M2-TAM compared to M1-TAM. The PPTT effect correlated with AuNPs uptake and was found to selectively target M2-TAM. The selective killing of protumerogenic M2-TAM while sparing anti-tumorigenic M1-TAM can hinder tumor development and contribute to the successful PPTT treatment of solid tumors. A better understanding of the role of TAM phenotype in PPTT will facilitate further optimization of the nanoparticle being used and guide future translational work.

## Experimental section

### Synthesis of gold nanorods

AuNRs were synthesized by seed-mediated growth following methods of Nikoobakht and El-Sayed.^18^ The seed solution was prepared as follow; 2.50 mL of HAuCl_4_ (1 mM) was mixed with 5 mL of cetyltrimethylammonium bromide (0.200 M, CTAB). 600 μL of ice-cold NaBH_4_ (10 mM) was added to the stirred solution and allowed to react for several minutes, until forming the pale brown gold seed solution. Next, the growth solution was prepared by adding 100 mL of HAuCl_4_ (1 mM) to 100 mL of CTAB (0.200 M) and 4.50 mL of AgNO_3_ (4 mM), followed by adding 1.40 mL of ascorbic acid (78.8 mM) was with gentle mixing to form the transparent growth solution. 160 μL of the seed solution was mixed with the unstirred growth solution and kept undisturbed for 2 hours. The resultant CTAB stabilized AuNRs were purified by centrifugation and redispersion in deionized (DI) water.

### Synthesis of gold nanocubes

Gold nanocubes (AuNCs) with an edge length of ∼45 nm were prepared by the seed-mediated method reported by Murphy *et al.*^19^ The seed nanoparticles were prepared by reduction of 2.75 mL HAuCl_4_·3H_2_O (0.909 mM), mixed with a solution of 0.283 g of CTAB dissolved in 5 mL DI water, by addition of 600 μL of an ice cold 0.01 M NaBH_4_ solution under stirring for 2 min. After 1 h, 0.35 mL of 10-fold diluted seed solution was allowed to grow for 4 h in a growth solution. The growth solution was prepared by mixing CTAB solution (2.916 g in 400 mL DI water) with HAuCl4·3H_2_O solution (0.0394 g dissolved in 143 mL DI water) followed by the addition of 6 mL ascorbic acid (1 M). The resulting CTAB stabilized AuNCs were purified by centrifugation and redispersion in DI water. The extinction coefficient of the AuNCs was estimated to 3.1 × 10^10^ M^−1^ cm^−1^.

### Preparation of PEG/RGD/NLS-functionalized gold nanoparticles

In order to reduce the cytotoxicity of the CTAB stabilized gold nanoparticles (AuNCs or AuNRs), the NPs were first modified with mPEG-SH to replace the CTAB. Here, 15 mL of 0.217 nm AuNCs was incubated with 85.7 μL of mPEG-SH (1 mM) for 24 h. For conjugating AuNRs with mPEG-SH, 10 mL of 1.6 nM AuNR suspension was treated with 29 μL of 1 mM mPEG-SH solution for 24 h. Afterward, the PEGylated nanoparticles were treated with cysteine-terminated RGD (RGDRGDRGDRGDPGC) and NLS (CGGGPKKKRKVGG) peptides at a ratio of 4 : 10 to yield PEG/RGD/NLS functionalized nanoparticles as described in detail in Panikkanvalappil *et al.*^20^ The nanoparticles at different stages of preparation were purified by centrifugation to remove unbound ligands. The extinction coefficient of the AuNRs was estimated to 1.4 × 10^9^ M^−1^ cm^−1^.

### Cell culture and differentiation

The human monocytic THP-1 cells were used for generating distinct subsets of macrophages phenotypes. The THP-1 cells were grown in RPMI-1640 medium (Gibco) supplemented with 10% fetal bovine serum (Gibco), and 1% penicillin/streptomycin (Gibco). For macrophages polarization experiments, the cells were seeded into a six-well culture plate (Sigma) and treated with phorbol myristate acetate (PMA, 200 ng mL^−1^) for 48 h to generate macrophages (M0). To generate M1-polarized macrophages, the obtained M0 macrophages were treated with 100 ng mL^−1^ LPS and 20 ng mL^−1^ IFN-γ for 24 h. To generate M2-polarized macrophages, the M0 macrophages were treated with 20 ng mL^−1^ IL-4 and 20 ng mL^−1^ IL-13 for 72 h.

### Flow cytometric analysis of biomarker expression

THP-1 cells were differentiated first to M0 then polarized to M1-Mfs or M2-Mfs, then the cells were blocked with 5 μl of Human TruStain FcX™ (Fc Receptor Blocking Solution, Biolegend) for 10 minutes at room temperature. Subsequently, M0 and THP-1 monocytes were stained with antibodies for CD68-FITC, while the M1 and M2-Mfs stained with anti-CD-86-PE-Texas Red or CD206-APC respectively or isotype control for 30 min in ice. For intracellular staining, M0 were first fixed and permeabilized for 45 min at 4 °C prior to staining with anti-CD68-FITC in permeabilization buffer. All antibodies were purchased from BioLegend and used at concentrations recommended by the supplier. Data analysis was performed on FlowJo software (TreeStar). The median fluorescence intensities (MFI) were calculated and plotted as fold difference as compared with the respective isotype controls.

### The dark-field imaging

The THP-1 cells were seeded at a density of 5 × 10^6^ on 18 mm glass coverslips in complete growth medium containing PMA, 100 ng mL^−1^ at 37 °C for 48 h. Afterwards, the cells were incubated with either 100 ng mL^−1^ LPS and 20 ng mL^−1^ IFN-γ or 20 ng mL^−1^ IL-4 and 20 ng mL^−1^ IL-13 for 24 h for generation of M1 and M2 macrophages, respectively. The different phenotypes of macrophages (M0, M1 and M2) were treated with 0.2 nM AuNCs@PEG/RGD/NLS diluted in supplemented clear culture medium, and the dark-field images were captured after the incubation time using a Leica microscope coupled with a Renishaw Via Raman microscope. ImageJ software was used for quantitative measurements of the light scattering intensities of dark-field images.

### Flow cytometry analysis of AuNPs cellular uptake

To study the cellular uptake using flow cytometry, the THP-1 cells were allowed to differentiate as mentioned before and treated with 0.2 nM AuNCs@PEG/RGD/NLS for 20 h. The differentiated treated cells were washed three times with Dulbecco's phosphate buffered saline (DPBS, Welgene, Korea), collected using cell scraper, centrifuged at 1500 rpm for 5 min, and resuspended in DPBS. Then, the suspended cells were analyzed using Fortessa FACS flow cytometry (Becton Dickinson, San Jose, CA, USA) equipped with a 488 nm argon laser. FlowJo software (TreeStar Inc) was used for further analysis of flow cytometry scattering data.

### Optical density measurement

To determine the amounts of cellular uptake of the gold nanoparticles, gold nanoparticles diluted in clear cell culture medium was incubated with the differentiated THP-1 cells in 12-well tissue culture plates for up to 20 h. The optical density of the collected media during and after the incubation period was measured using UV-vis spectroscopy and subtracted from the optical density of the initial media containing the tested nanoparticles.

### *In vitro* photothermal assay

The THP-1 cells were cultured in 12-well plates containing the RPMI medium supplemented with 10% FBS plus 100 ng mL^−1^ PMA for 48 h. Then the PMA-polarized Mfs (M0) were further treated with LPS/IFN-γ or IL-4/IL-13 to produce M1 and M2 polarized Mfs, respectively. Subsequently, M1 and M2-Mfs were treated with 0.2 nM AuNPs@PEG/RGD/NLS for 20 h, followed by washing with DPBS and NP-free medium was added. The cells were exposed to 808 nm NIR laser irradiation (1.0 W cm^−2^) for 2 min. For AuNRs, a separate set of M1 and M2-Mfs cells were also exposed to 808 nm NIR laser irradiation (1.0 W cm^−2^) for 2 min without removing the media containing the AuNRs. Then, the cells of the two sets were collected and evaluated for viability using an XTT assay and/or apoptosis/necrosis assay using Annexin-V/PI double staining in darkness for 15 min at room temperature, and analyzed by Fortessa Flow Cytometer (Becton Dickinson, San Jose, CA, USA). The apoptosis/necrosis assay was conducted following method described by Hala *et al.*^21^

### Cell viability using XTT assay

The XTT assay was performed according to the manufacturer's instructions (Biotium, Fremont, CA). Briefly, the cells were prepared in 96-well plates as mentioned in the *in vitro* photothermal experiment then cells were washed with PBS followed by treatment with XTT reagent diluted in clear DMEM. After this, the plate was incubated for 6 h at 37 °C in 5% CO_2_ incubator, then the optical density was measured at 450 and 690 nm in a BioTek Synergylabs H4 multimode plate reader.

### Statistical analysis

Statistical analysis was performed using statistical Package for Social Science (SPSS, IBM). Significant differences among means were evaluated using One-Way Anova Test. Results are expressed as mean ± SE. Probability values of less than 0.05 were considered significant.

## Results and discussion

### AuNPs synthesis, conjugation, and characterization

**Fig. 2:**
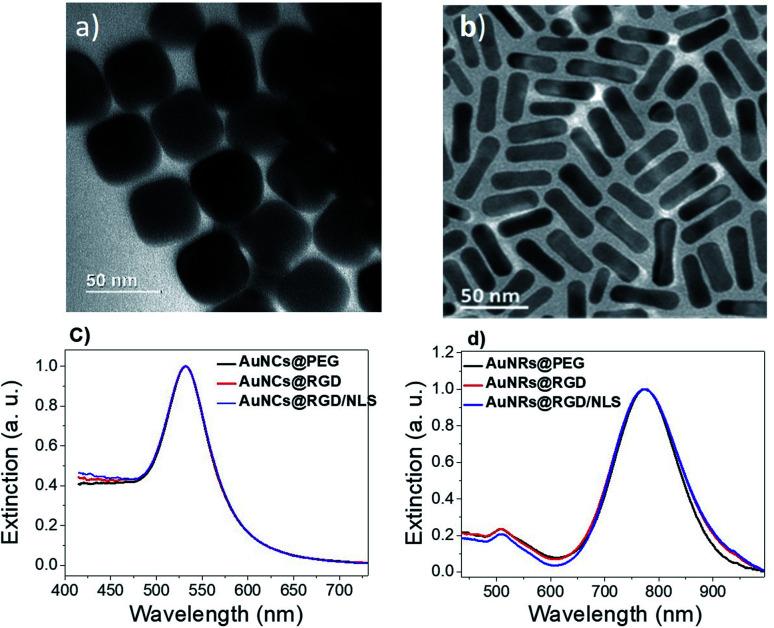
Transmission electron micrographs of (a) AuNCs and (b) AuNR. Corresponding UV-vis spectra of (c) PEGylated AuNCs (black, AuNC@PEG) and (d) AuNRs (black, AuNR@PEG), before and after conjugation with RGD (red, AuNPs@PEG/RGD) and RGD + NLS peptides (blue, AuNPs@PEG/RGD/NLS).

Two different shapes of AuNPs were synthesized (Fig. 2a, b and S1 ESI†). Gold nanocubes (AuNCs) were prepared by a modified seed mediated method reported by Murphy *et al.*^19^ Gold nanorods (AuNRs) were prepared by the method reported by Sajanlal *et al.*^20^ The average size of the AuNRs was ∼36 × 12 nm (aspect ratio: 3) and the average edge length for AuNCs was ∼45 nm. After synthesis, excess cetyltrimethylammonium bromide (CTAB) was removed by centrifugation. Remaining CTAB was replaced by mPEG-SH by ligand–ligand exchange in order to improve colloidal stability and reduce unspecific protein adsorption.^22^ The PEGylated nanoparticles were then incubated with the Cys-containing RGD peptide to generate AuNPs@PEG/RGD or combinations of RGD and NLS peptides at a ratio of 4 : 10 to produce AuNPs@PEG/RGD/NLS, to promote TAM uptake. The changes in the localized surface plasmon resonance (LSPR) band of the nanoparticles upon functionalization was monitored using ultraviolet-visible (UV-vis) spectroscopy. Peptide-functionalization did not result in any obvious shift in the LSPR band of the AuNCs (*λ*_max_ = 530 nm), likely due to the low molecular weight of the peptides (Fig. 2c). Because of the higher RI sensitivity of the AuNRs, a slight LSPR redshift could be seen for the longitudinal LSPR band of the AuNRs, from about 773 to 774 nm, after peptide conjugation (Fig. 2d).

Efficient and selective delivery of nanoparticles to target cancer cells and tumors has previously been reported in numerous studies by functionalization of the nanoparticles with RGD and NLS peptides.^23,24^ Peptides with the RGD motif are known to target cell surface integrins, which are overexpressed by most types of cancer cells as well as M2 TAM, enabling internalization of NPs into the cells *via* receptor-mediated endocytosis. The nuclear localization signal (NLS) peptide is targeting the cell nucleus by recognizing and binding to nuclear transport receptors. Because of the effects of RGD and NLS in targeting cancer cells and tumors,^25,26^ RGD/NLS-functionalized AuNPs were hence used in this work to promote AuNP uptake to investigate how macrophage polarization might impact plasmonic photothermal therapy.

### Human monocytic THP-1 differentiation and polarization

**Fig. 3:**
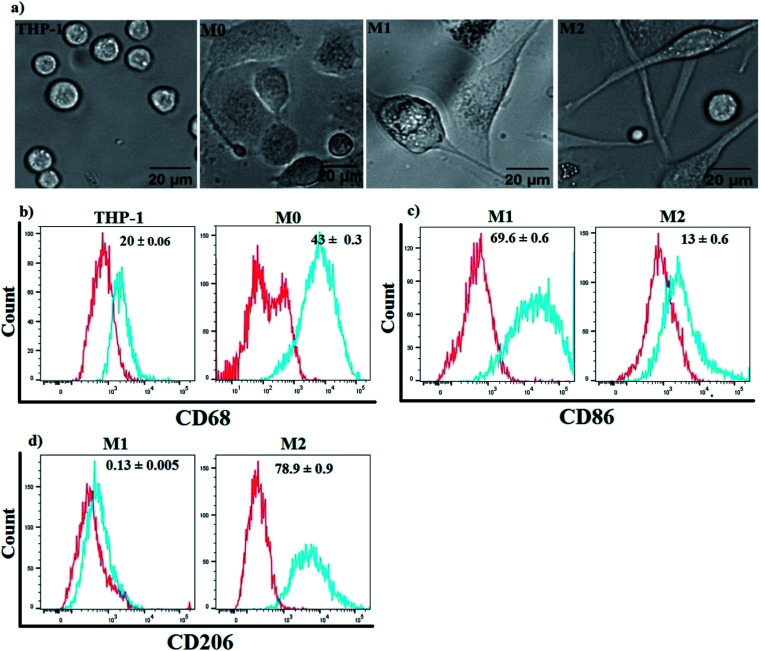
Differentiation of THP-1 human monocytic cells to M0 using PMA followed by LPS/INF or IL-4/IL-13 to induce M1 or M2, respectively. (a) The morphology of PMA-stimulated THP-1 (M0) show adherent cells with a slightly rounded shape while the polarized M1 and M2 macrophages show spindle shape morphology. (b–d) Flow cytometry histograms for surface expression of CD68, CD206, and CD86. Red line: isotype control; blue line: specific staining. One representative histogram is shown. Median of fluorescence intensity (MFI) values is given within graphs and represents the mean ± SE obtained from three independent experiments.

Three subtypes of Mfs (M0, M1 and M2) were generated from human monocytic THP-1 cells. Suspended THP-1 cells were treated for 48 h using phorbolmyristate acetate (PMA) followed by 24 h in PMA-free media to induce differentiation into M0-Mfs. The procedure resulted in cells with morphological characteristics related to Mfs, including adhesion, changes in size and shapes. Subsequently, M0-Mfs were stimulated with LPS/IFN-γ for 24 h or IL-4/IL-13 for 72 h to induce polarization into M1 and M2, respectively. The shape of M1-Mfs and M2-Mfs were spindle-like with differences in morphology between the two subtypes (Fig. 3a). The differentiation of the THP-1 human monocytic cells to M0-Mfs was confirmed by assessing the expression of the recognized macrophage marker CD68 (ref. 27) using flow cytometry (Fig. 3b). To evaluate the polarization strategy, the surface expression of M1 and M2 phenotypic markers CD86 and CD206,^28^ respectively, were analyzed using flow cytometry. The M1-Mfs exhibited clearly increased CD86 expression along with decreased CD206 expression while M2-Mfs expressed higher level of CD206 and low levels of CD86 (Fig. 3c and d).

### Cellular uptake of polarized macrophages enhances the PPTT performance

**Fig. 4:**
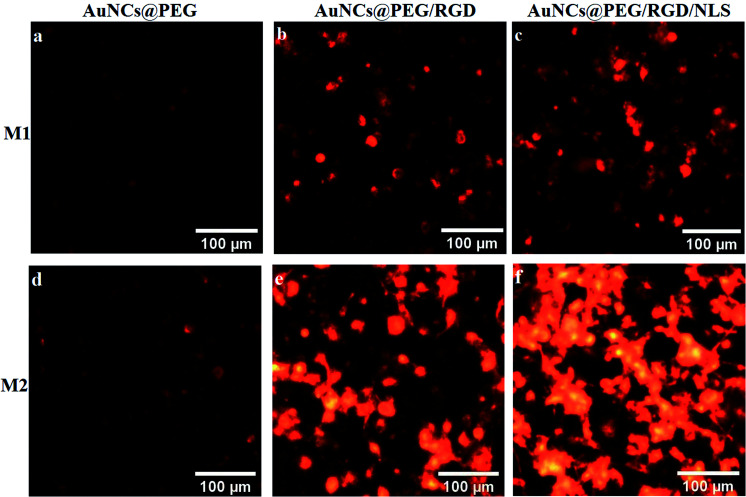
Dark field imaging of two differently polarized macrophages representing (a–c) M1-TAM and (d–f) M2-TAM after 20 h treatment with 0.2 nM of PEGylated AuNCs or PEGylated AuNCs functionalized with RGD or RGD/NLS.

To explore the role of Mfs plasticity in modulating AuNP uptake, the two polarized subsets, M1-Mfs and M2-Mfs, were exposed to 0.2 nM of PEGylated AuNCs functionalized with RGD or RGD/NLS. The efficiency of nanoparticle uptake was first investigated using dark field microscopy. The M2-Mfs show higher light scattering intensity compared to M1-Mfs after treatment (Fig. 4 and S2 ESI†), suggesting that cellular uptake of AuNPs was significantly affected by the macrophage phenotype. The dark field images indicated that M2-Mfs exhibit greater uptake for both the RGD and RGD/NLS-functionalized AuNCs compared to just PEGylated AuNCs, indicating that RGD and NLS functionalization enhances the internalization of the nanoparticles.

**Fig. 5:**
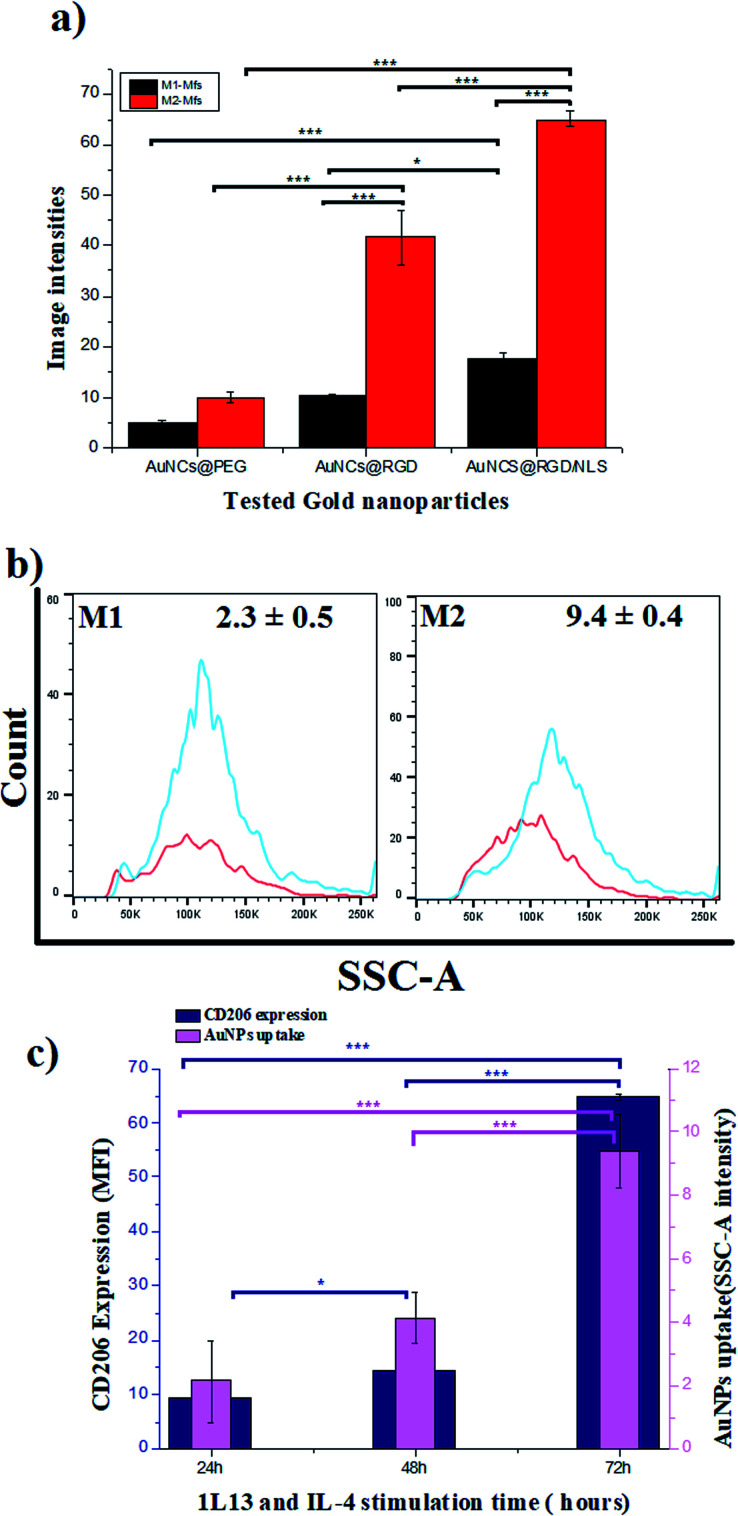
Quantitative measurement of the cellular uptake of M1-Mfs and M2-Mfs exposed to 0.2 nM PEGylated RGD/NLS-functionalized AuNCs based on (a) dark field image intensities, and (b) flow cytometry side scattering intensity histograms. (c) Correlation between CD206 protein expression and uptake of PEG/RGD/NLS-functionalized AuNCs. Results are expressed as means ± and *P* value <0.05 are considered significant. *P < 0.05 and ***P < 0.0001.

To quantify the cellular uptake, the light scattering intensities of the dark field images were determined. The light scattering showed a 2-, 4- and 3.7-fold increase in M2-Mfs uptake in comparison to M1-Mfs for AuNCs@PEG, AuNCs@PEG/RGD and AuNCs@PEG/RGD/NLS, respectively (Fig. 5a). The RGD peptide sequence enhances endocytotic uptake *via* integrin binding. Subsequently, the nuclear targeting peptide, NLS, facilitate particle delivery and accumulation within the nucleus.^29^ However, Oh *et al.* recently showed that PEGylated nanoparticles that are endocytosed by M1 or M2 macrophages, to a large extent are exocytosed.^30^ Within 24–48 hours up to 60% of the nanoparticles were exported out of the cells. Due to the NLS peptides, this process is likely less pronounced which supports the finding that a larger fraction of the AuNCs@PEG/RGD/NLS are retained in the cells as compared to AuNC@PEG and AuNCs@PEG/RGD. This trend was further confirmed by flow cytometry. Flow cytometry was used to quantify the uptake of AuNCs@PEG/RGD/NLS by M1-Mfs and M2-Mfs using the side scattering (SSC) intensities of treated cells compared to untreated cells. The SSC intensities varied with respect to the Mfs phenotype and the M2-Mfs showed about 4-fold increase in SSC intensity as compared to treated M1-Mfs (Fig. 5b and c). The flow cytometry result was hence in good agreement with the quantitative analysis of light scattering intensities of AuNPs-exposed Mfs from dark field microscopy images. The effectiveness of flow cytometry in measurement of cellular uptake of nanoparticles with SSC is well documented in previous studies.^31^ For instance, Park *et al.* compared SSC intensities of HeLa cells exposed to different types of AuNPs (40–100 nm) with the amount of intracellular AuNPs measured by inductively coupled plasma mass spectrometry (ICP-MS) and found a linear correlation between the concentration of internalized AuNPs and the SSC intensities.^32^ Zucker *et al.* confirmed the relationship between flow cytometry SSC and cellular TiO_2_ NPs and AgNPs from dark field images.^33,34^ Moreover, the UV-vis absorbance of AuNCs@PEG/RGD/NLS in the cell culture media was recorded before and after 8 h incubation with M1-Mfs and M2-Mfs and showed a larger decrease in concentration of suspended nanoparticles in the media of M2-Mfs compared to M1-Mfs, further suggesting that M2-Mfs internalized higher amounts of the nanoparticles (Fig. S3 ESI†). No visible nanoparticle precipitates were observed in the cell culture media and the UV-vis spectra showed no, or only minor, shifts in the LSPR bands after 20 hours in the absence of cells, indicting colloidally stable non-aggregated nanoparticles.

RGD-modified nanomaterials have recently been shown to enhance the polarization of macrophages towards the M2 phenotype while blocking the polarization into the M1 phenotype.^35^ More importantly, β-1 and β-2 integrins have been found to be strongly expressed in IL-4 induced macrophages.^36^ This might explain the preferential uptake of RGD functionalized nanoparticles by the M2-TAM over M1-TAM. Moreover, the greater endocytic capacity of polarized M2-TAM has been linked to increased expression of receptors facilitating endocytosis, such as scavenger and mannose receptors.^37,38^ For example, Orr *et al.* demonstrated that silencing expression of scavenger receptor A inhibited the uptake of amorphous silica nanoparticles by macrophage cells.^39^ Our data indicate a clear correlation between the surface marker expression of CD206 and AuNCs uptake. Both CD206 expression and AuNCs uptake increase with time of stimulation of the M0 with IL-4 and IL-13 (Fig. 5c and S4 ESI†). CD206 expression is more pronounced in M2-Mfs compared to M1-Mfs, suggesting that high CD206 expression in M2-Mfs correlate with their higher capacity to take up the nanoparticles. This finding is consistent with a previous study demonstrating that the expression of typical M2-Mfs surface receptors (CD163, CD206) show positive correlation with uptake of 100 nm AuNPs.^40^ However, more work is needed to fully confirm the potential relationship between CD206 expression and M2-Mfs uptake capacity. M2-TAM is considered to be a polarized M2-like macrophage that can reprogram the TM by secreting immunosuppressive factors that influence the progression and spreading of tumors. High density of M2-TAM in solid tumors is highly associated with a poor patient prognosis. Therefore, targeting M2-TAM and eliminating their support for tumor growth has been previously addressed as an approach to improve tumor treatment.^41,42^ Based on these previous attempts, we argue that the augmented AuNP uptake by M2-Mfs in comparison to M1-Mfs contribute to improve the PPTT efficacy in tumor therapy.

**Fig. 6:**
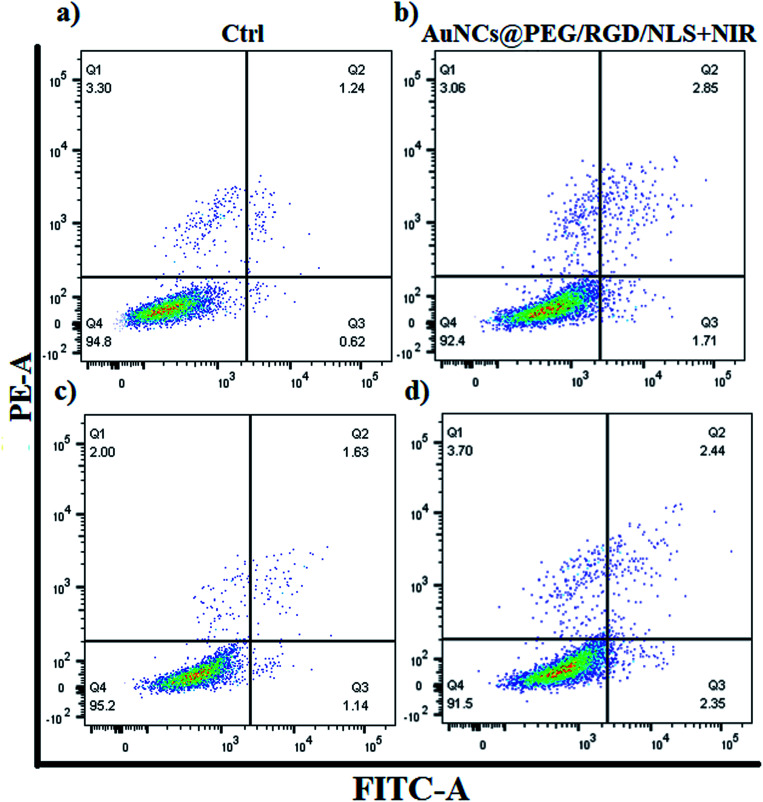
The effect of AuNCs-assisted plasmonic photothermal heating on cell viability of M1-Mfs and M2-Mfs assessed using apoptosis/necrosis assay and flow cytometry. (a) M1-Mfs (untreated control) and (b) M1-Mfs treated with AuNCs@PEG/RGD/NLS + NIR laser. (c) M2-Mfs (untreated control) and (d) M2-Mfs treated with AuNCs@PEG/RGD/NLS + NIR laser.

In order to investigate if the preferential AuNP-uptake by M2-Mfs can be translated into efficient and selective photothermal heating mediated killing of M2-TAM, the two polarized Mfs subsets (M1-Mfs and M2-Mfs) were treated with 0.2 nM AuNCs@PEG/RGD/NLS. After 20 h incubation, the cell culture media containing the NCs was replaced with fresh nanoparticle-free medium followed by 2 minutes of 808 nm CW NIR laser irradiation. The cell viability was then measured using flow cytometry. The result showed a slight decrease in viability of about 2.4 and 3.7% for M1-Mfs and M2-Mfs, respectively (Fig. 6 and S5 ESI†). This result indicates that photothermal effect caused by RGD/NLS-functionalized AuNCs@PEG was not sufficient to kill the cells, likely because of the relatively poor photothermal properties of AuNCs at this wavelength since the localized plasmon resonance does not match with the laser wavelength (808 nm). Intracellular aggregation of AuNCs could shift the plasmon band towards higher wavelength through interplasmon coupling,^43^ but the heating efficiency is still very low compared to AuNRs. However, their pronounced light scattering makes them very suitable for imaging (Fig. 4). In contrast, AuNRs demonstrate more prominent photothermal properties with a distinct absorption of light in the near infrared (NIR) wavelength range.^44,45^

The efficient NIR-induced photothermal effect of AuNRs have been successfully applied for tumor treatment because of the large tissue penetration depth of NIR light.^46^ Therefore, AuNRs with the identical surface functionalization as the AuNCs were used here to further explore the effect of PPTT for selectively targeting the M2-TAM. In this experiment the two polarized Mfs (M1-Mfs and M2-Mfs) were incubated with 0.2 nM AuNRs@PEG/RGD/NLS for 20 h followed by 2 minutes of NIR laser exposure under two different conditions. The first condition was aiming at exposing the cells to NIR laser in the presence of extracellular AuNRs in the cell culture media. Under the second condition, the culture media containing AuNRs was replaced with AuNR-free media before laser exposure. The rationale was to elucidate the influence of the uptake rate on the photothermal effect and excluding the photothermal effect arising from heating of any extracellular particles.

**Fig. 7:**
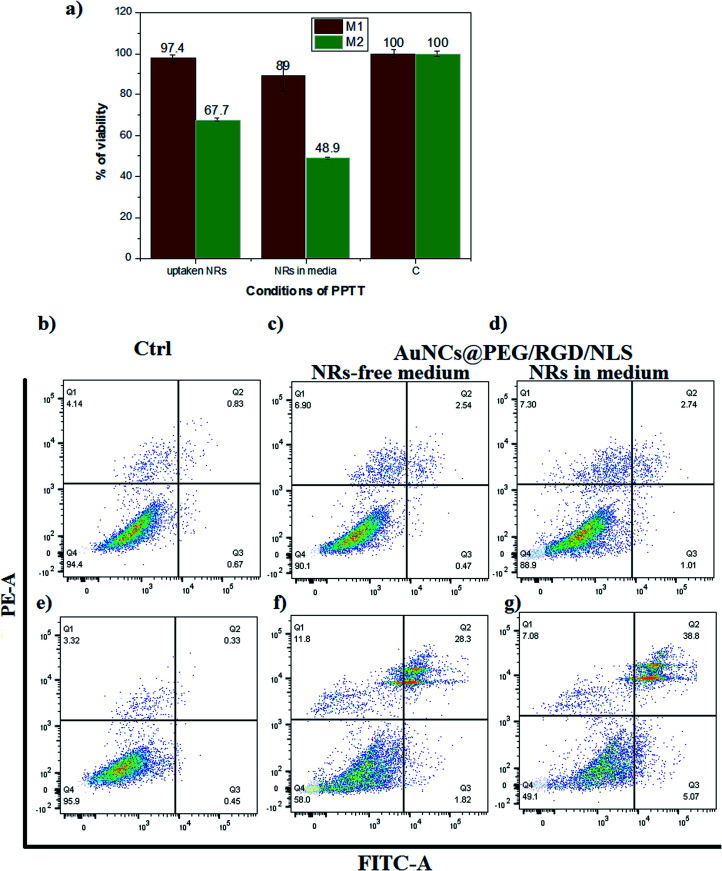
The effect of AuNR-assisted plasmonic photothermal heating on cell viability of M1-Mfs and M2-Mfs. (a) XTT cell viability assay (*C* = negative control). Flow cytometry experiment for characterizing apoptosis/necrosis: (b) M1-Mfs (untreated control), (c) M1-Mfs treated with AuNRs@PEG/RGD/NLS + NIR laser in AuNR-free medium, (d) M1-Mfs treated with AuNRs@PEG/RGD/NLS + NIR laser in AuNR-containing medium, (e) M2-Mfs (untreated control), (f) M2-Mfs treated with AuNRs@PEG/RGD/NLS + NIR laser in AuNR-free medium, (g) M2-Mfs treated with AuNRs@PEG/RGD/NLS + NIR laser in AuNR-containing medium.

The viability of the cells after irradiation was investigated using an XTT assay (Fig. 7a). The M1-Mfs viability was almost unaffected by laser irradiation, reaching viabilities as high as 97.4% and 89% for the two different conditions.

In contrast, the viability of the M2-Mfs was reduced to 67.7% and 48.9% for the same two conditions. The results of the XTT assay were verified using an apoptosis necrosis assay which indicated that M2-Mfs were more susceptible to apoptotic cell death after PPTT than M1-Mfs (Fig. 7b–g). The percentage of M2-Mfs viable cells was reduced from 95.9% in the untreated control to 58% under the first condition and 49.1% under the second condition. In contrast, the percentage of M1-Mfs viable cells was 94.6% in the control and only slightly reduced to 90.1% and 88.9% under the first and second condition, respectively. Bright field imaging of both M1 and M2-Mfs show a pronounced change from spindle like to rounded cells for the latter after laser treatment and nanoparticle uptake (Fig. S6 ESI†). These results correlate with our previous findings that the effects of nanoparticles alone without laser irradiation or irradiation without nanoparticles result in very low cell death of MCF-7 cells in comparison to when combining nanoparticles and irradiation with a laser.^17^ Moreover, a 2-minutes exposure of mammary gland tumors in canine and feline after gold nanoparticle uptake by an 808 nm diode laser with a power of 5.8 W cm^−2^ and a spot size of around 5.6 mm^2^ resulted in heating to 42–44 °C, which is associated with apoptotic cell death.^17^ Here, the drastic increase in cell death of M2-Mfs suggests that their pronounced uptake of PEGylated and RGD/NLS functionalized gold nanoparticles combined with the efficient heat generation of the AuNRs upon laser irradiation enables selective PPTT targeting of the protumoral M2-Mfs.

Altogether, the current findings indicate that efficiency in using PPTT for treatment of solid tumors could partly be due to the selective targeting of M2-TAM in the TM while leaving the M1-TAM largely unaffected.

## Conclusions

In conclusion, the amount of cellular uptake of AuNPs correlates with the effect of plasmonic photothermal heating upon NIR laser exposure. Therefore, the observed higher rate of AuNPs uptake by M2 compared to M1 polarized macrophages can be translated into selective laser heating and killing of protumoral M2-Mfs without damaging the anti-tumoral M1-Mfs. This finding can have a positive impact on the use and optimization of PPTT in combating tumors as it can enable complete eradication of the protumoral cells and eliminate their support to malignant cancer cells. The selective killing of M2-TAM can potentially improve the response of tumors to treatment. In general, these observations may open up new avenues for development of novel therapeutic interventions based on selective targeting of protumoral M2-Mfs and consequently block tumor progression. Further studies in relevant tumor models should be conducted prior advancing this technology to clinical trials.

## Author contributions

The manuscript was written through contributions of all authors. All authors have given approval to the final version of the manuscript.

## Conflicts of interest

There are no conflicts to declare.

## Supplementary Material

RA-011-D1RA03671H-s001
