# Supplementary material for: Effects of macrophage polarization on gold nanoparticle-assisted plasmonic photothermal therapy
Source: RSC Adv. 2021 Jul 19;11(40):25047–56. doi: 10.1039/d1ra03671h (PMC9037012; doi:10.1039/d1ra03671h)
Supplement: RA-011-D1RA03671H-s001 [file RA-011-D1RA03671H-s001.pdf]

Electronic Supplementary Material (ESI) for RSC Advances.
This journal is © The Royal Society of Chemistry 2021

Electronic Supplementary Information

Effects of Macrophage Polarization on Gold Nanoparticle-Assisted Plasmonic Photothermal Therapy

Hala R. Ali,[a]* Salah A. Selim[b] and Daniel Aili[c]*

[a]Department of Bacteriology and Immunology, Animal Health Research Institute (AHRI), Agriculture Research Center (ARC), Dokii-Giza, P.O. Box12618, Egypt.

[b]Department of Microbiology, Faculty of Veterinary Medicine, Cairo University, Giza, Cairo, Egypt.

[c]Laboratory of Molecular Materials, Division of Biophysics and Bioengineering, Department of Physics, Chemistry and Biology, Linköping University, SE-581 83 Linköping, Sweden.

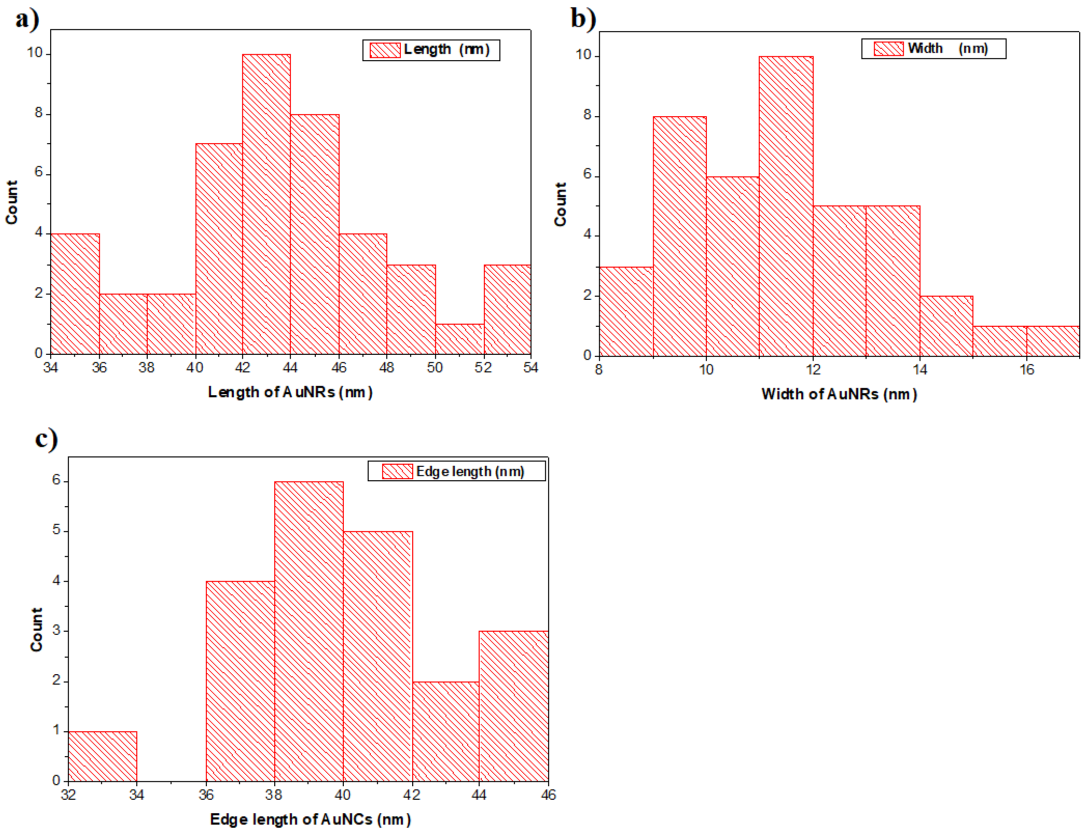

Fig. S1. Size distributions of the nanoparticles used. a) Length and b) width of the AuNRs, and c) edge length of AuNCs.

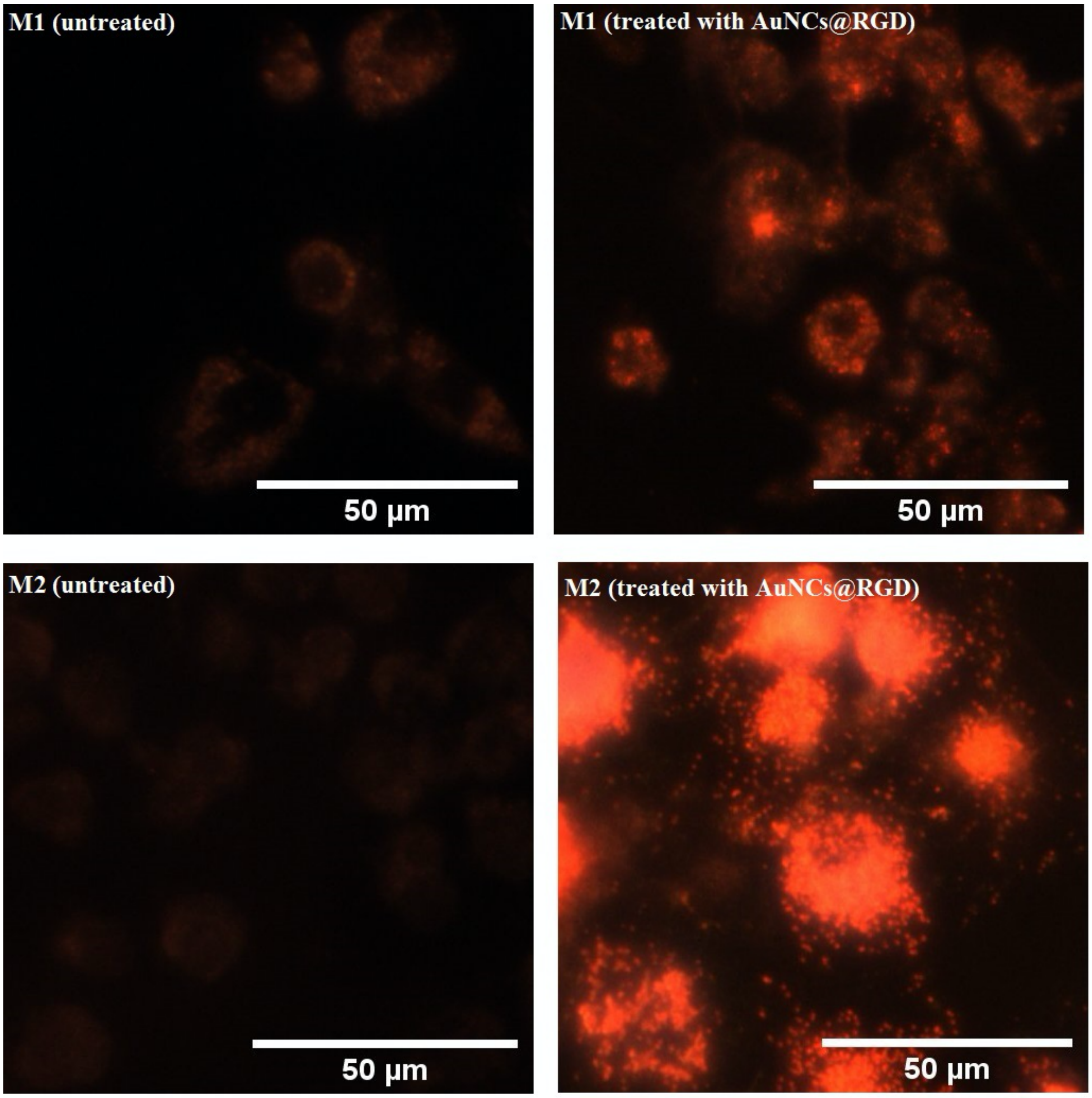

Fig. S2. Dark field images of M1 and M2 macrophages treated with 0.2 nM AuNCs@PEG/RGD.

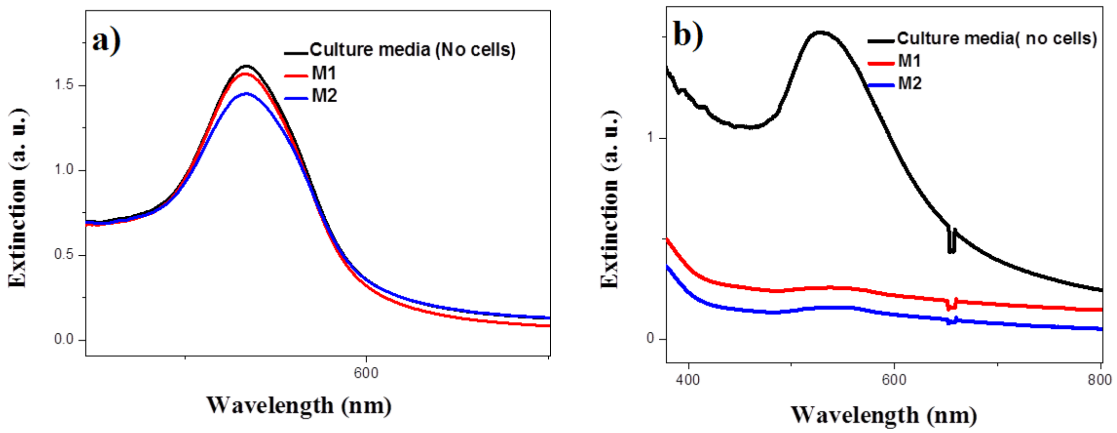

Fig. S3. UV-vis spectra of AuNCs@PEG/RGD/NLS in cell culture media before and after a) 8 hr and b) 20 hr incubation with M1 and M2 macrophages.

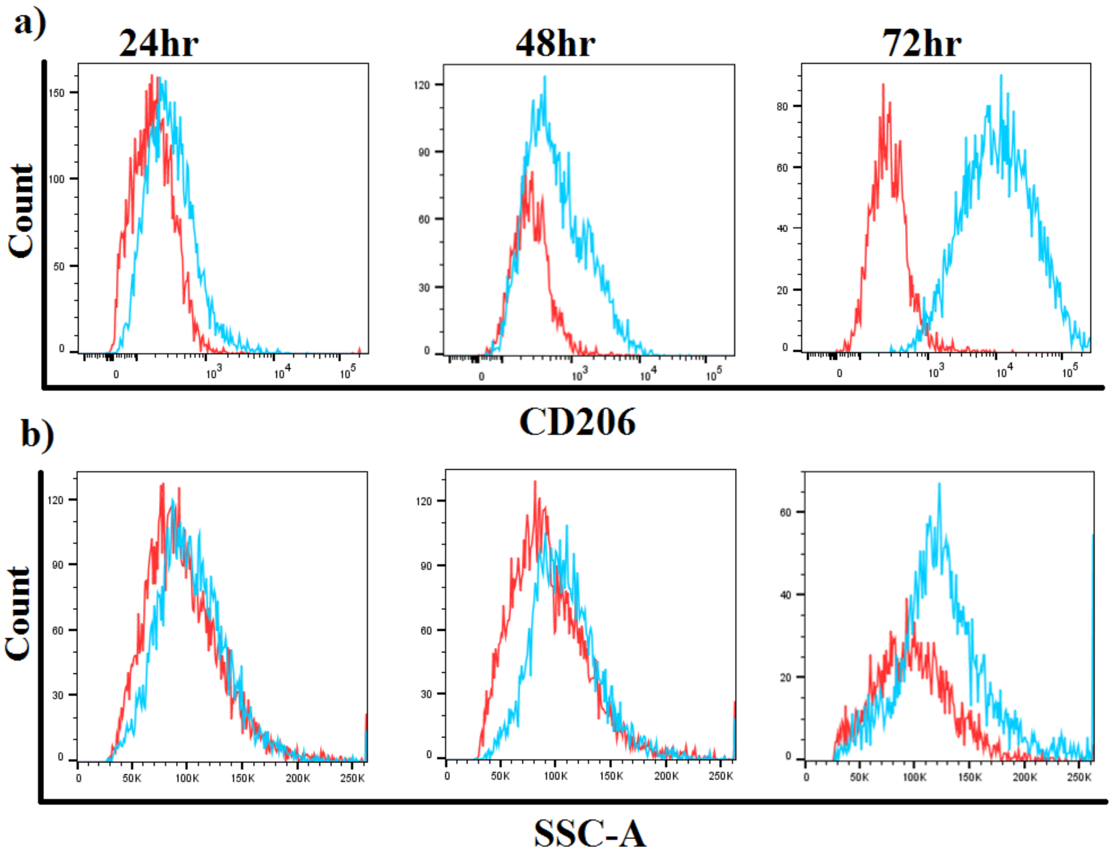

Fig. S4. Correlation between the CD-206 expression and cellular uptake of AuNCs@PEG/ RGD/NLS. a) CD206 expression after stimulation of M0 with IL-4 / IL13 for 24, 48 and 72 hr. b) Histogram of THP-1 macrophages exposed to AuNCs after stimulation with IL-4 / IL13 for 24, 48 and 72 hr.

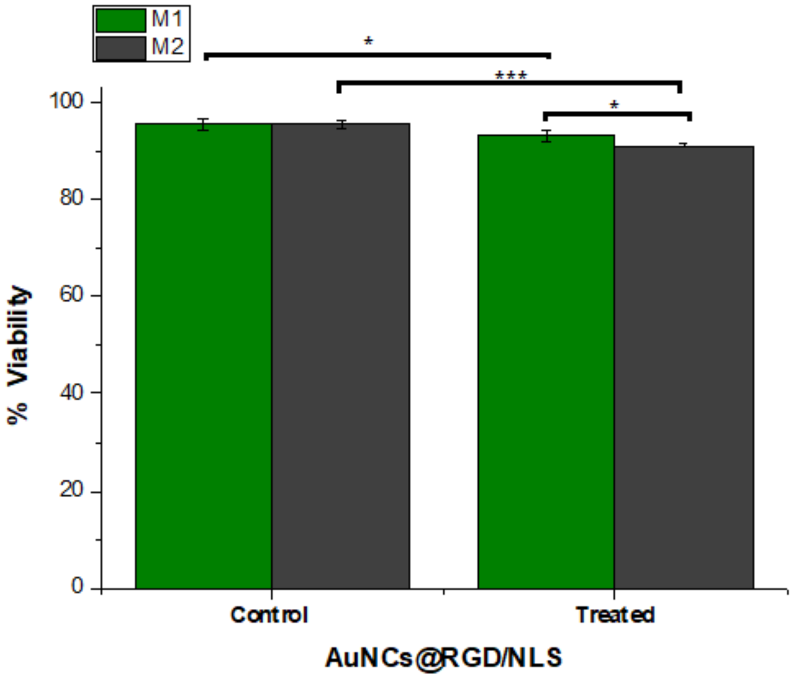

Fig. S5. Viability % of M1 and M2 macrophages upon treatment with AuNCs assisted plasmonic photothermal therapy. The percentage of viable cells is measured by apoptosis/ necrosis assay. Results are expressed as means ± and P value < 0.05 are considered significant. ***: P <0.0001 & *: P<0.05.

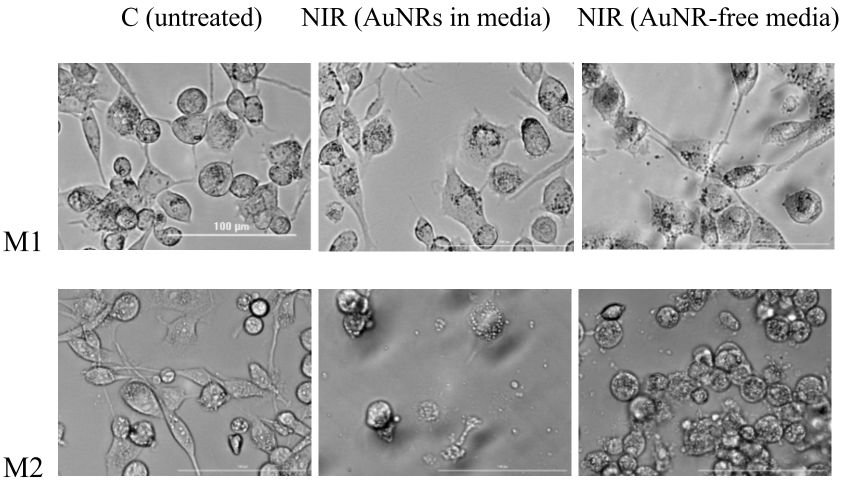

Fig. S6. Bright field images of M1 and M2 macrophages before and after AuNR-assisted plasmonic photothermal therpy in prescence or absence of extracellular RGD and NLS functionalize PEGylated AuNRs. Scale bare is 100 µm.
